# Presentation, Molecular Characteristics, Treatment, and Outcomes of Colorectal Cancer in Patients Older than 80 Years Old

**DOI:** 10.3390/medicina59091574

**Published:** 2023-08-29

**Authors:** Ioannis A. Voutsadakis

**Affiliations:** 1 Algoma District Cancer Program, Sault Area Hospital, 750 Great Northern Road, Sault Ste. Marie, ON P6B 0A8, Canada; ivoutsadakis@yahoo.com or ivoutsadakis@nosm.ca; 2 Section of Internal Medicine, Division of Clinical Sciences, Northern Ontario School of Medicine, Sudbury, ON P3E 2C6, Canada

**Keywords:** gastrointestinal, geriatric population, adverse effects, toxicity, disparities

## Abstract

*Background*: An increasing proportion of the population of patients with cancer presents at an advanced age, increasing the challenges of successful and well-tolerated treatments. In the older spectrum of the geriatric cancer patients, those older than 80 years old, challenges are even higher because of increasing comorbidities and decreasing organ function reserves. *Methods*: Studies regarding colorectal cancer presentation, treatment, and prognosis in patients older than 80 years old available in the literature were evaluated and were compiled within a narrative review. Molecular attributes of colorectal cancer in the subset of patients older than 80 years old in published genomic cohorts were also reviewed and were compared with similar attributes in younger patients. *Results*: Characteristics of colorectal cancer in octogenarians are in many aspects similar to younger patients, but patients older than 80 years old present more often with right colon cancers. Surgical treatment of colorectal cancer in selected patients over 80 years old is feasible and should be pursued. Adjuvant chemotherapy is under-utilized in this population. Although combination chemotherapy is in most cases not advisable, monotherapy with fluoropyrimidine derivatives is feasible and efficacious. *Conclusions*: Outcomes of colorectal cancer patients over the age of 80 years old may be optimized with a combination of standard treatments adjusted to the individual patient’s functional status and organ reserves. Increased support for the older age group during their colorectal cancer treatment modalities would improve oncologic outcomes with decreasing adverse outcomes of therapies.

## 1. Introduction

Cancer is commonly a disease of the old, and median age at diagnosis considering all disease sites is 65 years according to U.S. Surveillance, Epidemiology and End Results (SEER) data [1]. Cancer registry data from Canada showed that 16% of cancer patients aged 45 to 99 years old were diagnosed at age 80 or older [2]. Several common cancers such as lung carcinomas, prostate cancer, and gastrointestinal carcinomas have a median age at presentation above 65 years old [3]. In 2018, colon and rectal cancers accounted for over 1.8 million cases worldwide and 860,000 deaths. Improvements in survival relating to metastatic colorectal cancer have been accomplished in recent decades with incremental benefits obtained by the introduction of more effective chemotherapies, targeted therapies, and a decreasing mortality trend. Colorectal cancer presents at a median age of 66 and 54% of patients are diagnosed with colorectal cancer at age above 65 years [4]. Patients older than 80 years old constitute a significant percentage of geriatric colorectal cancer patients. The incidence of colorectal cancer increases from 90.2 per 100,000 population at age 60 to 64 to 237.9 per 100,000 population at age 80 to 84. The older age group is associated with greater challenges in treatment because of comorbidities and frailty, which become more frequent in the older population [5]. Studies have also observed that diagnosis may be delayed due to reduced screening, as guidelines advocate for selective screening after age 75 and discourage screening in patients older than 85 [6].

All treatments including surgery, adjuvant chemotherapy, and systemic therapies for metastatic disease are more challenging in older patients. This leads to the belief that standard treatment of cancers may not be appropriate in patients above age 80, and many older patients are not even referred for surgical, medical oncology or radiation oncology consultations [2]. However, it has been recognized from the beginning of the modern era of cancer therapy that age alone should not be the decisive factor for altering the recommended treatment strategy [7]. Challenges affecting surgical treatment and outcomes include cardiovascular and cerebrovascular comorbidities that increase the risks of general anesthesia, delayed healing of surgical wounds, and increased rates of complications.

Older patients may also present increased risk for adverse effects of chemotherapy treatment. Many patients, especially in the older part of the age spectrum (>85 years old), would not be considered for chemotherapy treatment by most oncologists, even when an oncologic indication exists, because of a perceived or actual increase in adverse effects of such therapy [8]. Elderly patients may also be considered more reluctantly as metastasectomy candidates for limited metastatic disease, for reasons similar to those that increase the risk of primary surgical treatment. These challenges could overall lead to inferior survival outcomes of colorectal cancer in elderly patients.

This article describes colorectal cancer presentation, biology, treatment, and outcomes in patients older than 80 years old. Improved tolerability of individualized treatments and better-tailored therapies that take into consideration the functional status and organ reserves of older patients could lead to optimized outcomes [9].

## 2. Presentation, Characteristics, and Biology of Colorectal Cancer in Older Patients

Clinicopathologic studies have addressed the presentation and characteristics of colorectal cancer in the older population [10,11,12]. Colorectal cancer patients in the older population (older than 80 years old) had a higher rate of comorbidities and lower performance status than younger patients, 60 to 80 years old [10]. In this series, there were no differences in the tumor grade, the presence of lymphovascular or perineural infiltration, the presence of microsatellite instability, the presence of elevated Carcinoembryonic Antigen (CEA), the number of lymph nodes excised, the location of the primary cancer in the colon, or the presentation as synchronous or metachronous disease between the two age groups of patients (Table 1). A study from the Los Angeles County Cancer Surveillance Program reported that patients over 80 years old represented 26% of the patients diagnosed with colorectal cancer in an 18-year period [11]. Women represented 60% of patients in the over 80-year-old cohort, while in younger patients the distribution of colorectal cancers was equal between sexes. Right colon cancers were more prevalent (65%) in the older cohort, while in the younger cohorts they represented 49% to 58% of cases (Table 1). Stage and tumor grade distributions were similar in the different age groups. In a similar study based on the Japanese Society for Cancer of Colon and Rectum registry that categorized patients according to their age group, 9.1% of patients were over 80 years old [12]. Cancers in patients over the age of 80 were more often (43% of cases) in the right colon, while in younger patients 27.8% of cancers were in the right colon. Older patients had an elevated CEA in 46.2% of cases, while an elevated CEA was observed in 40.8% of younger patients. Earlier stage cancers (stages I and II) were more frequent in patients over the age of 80 years old (56.8% of all cancers in this age group) than in patients younger than 80, where stage I and II cancers constituted 51.6% of all cancers [12].

The higher prevalence of right colon cancers in patients older than 80 years old was also observed in a series from Boston, where 73.3% of colon cancers in older patients were right-sided while 55.7% of cancers in patients 50 to 80 years old were right-sided (*p* < 0.001, Table 1) [13]. Octogenarians presented with larger primary tumors than younger patients but their disease was less often lymph node positive (36.8% versus 44.3% in the younger group, Fisher exact test *p* = 0.02) and less often metastatic (6.1% versus 17.9% in the younger group, Fisher exact test *p* < 0.001). In the California Cancer Registry, patients over 80 years old had more often undergone right colectomies than younger patients, suggesting a higher prevalence of right colon cancer in the old [15].

Compared with colorectal cancer patients younger than 80 years old, octogenarian colorectal cancer patients seen over a 5-year period in a center in Israel were less often diagnosed by screening and had less often a family history of colorectal or other types of cancer [14]. In contrast, octogenarians had a higher rate of personal history of cancer and more commonly a right colon cancer primary (45.7%) than younger patients who had right colon primaries in 34.3% of cases (*p* = 0.02). Older patients less often had good performance status (Eastern Cooperative Oncology Group Performance Status (ECOG PS) 0 or 1 in 71% of patients versus 93.9% in patients younger than 80, *p* < 0.001) and had more often received no treatment at all or received less intensive treatment [14].

**Figure 1:**
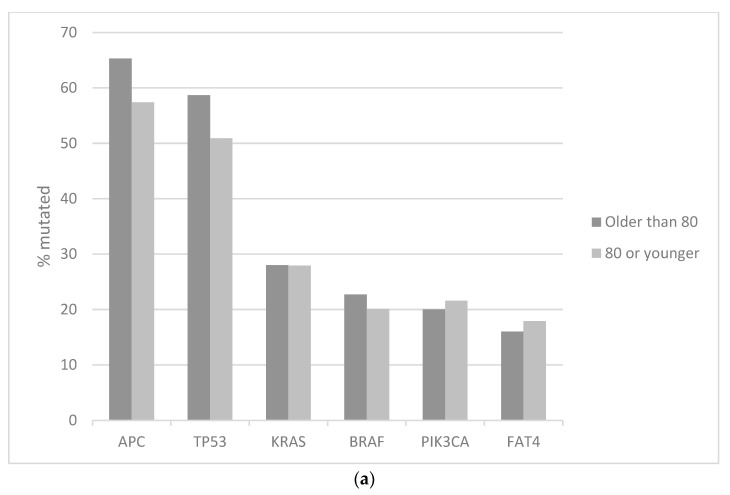

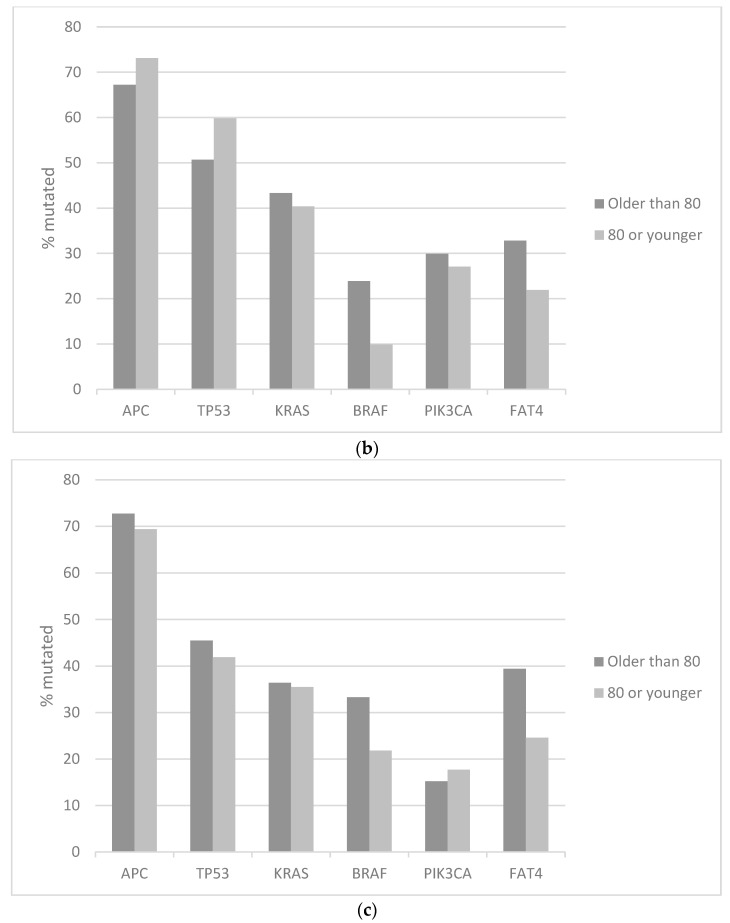
Prevalence of most frequent colorectal cancer mutations in patients older than 80 years old (black bars) and 80 years old or younger (grey bars). (**a**) Data from the DFCI (Dana Farber Cancer Institute) series. (**b**) Data from TCGA (the Cancer Genome Atlas) colorectal cancer study. (**c**) Data from the Sidra-LUMC AC-ICAM cohort.

In the genomic colorectal cancer series published by the Dana Farber Cancer Institute (DFCI) including over 600 patients, patients above the age of 80 years old represented 12.2% of the population [16]. Right colon primaries were more prevalent in patients older than 80 years old in this series (66% of patients) versus younger patients where right colon cancers were observed in 49% of cases (Fisher’s exact test *p* = 0.01). In contrast, there were no significant differences between the older and younger patients in the prevalence of metastatic disease, which was present in 14.9% of patients older than 80 years old and 11.1% of younger patients (Fisher’s exact test *p* = 0.4). The prevalence of poorly differentiated histology (9.3% in patients older than 80 years old and 8.3% in the younger patient group) was not significantly different between the groups (Fisher’s exact test *p* = 0.7). There were also no differences in the prevalence of microsatellite instability (13.2% in older and 17.7% in younger patients, Fisher’s exact test *p* = 0.5), and the prevalence of tumors with high levels of CpG islands methylation (22.5% in older and 18.5% in younger patients, Fisher’s exact test *p* = 0.5). Mutations in the most frequently mutated oncogenes and tumor suppressors in colorectal cancer, such as *APC*, *TP53*, *KRAS*, *BRAF*, and *PIK3CA* showed no significant differences between the group of colorectal cancer patients older than 80 years old and their younger counterparts (Figure 1a) [16].

In the colorectal cancer genomic series from The Cancer Genome Atlas (TCGA), which included more than 500 patients, 13% of patients were over the age of 80 years old [17]. This series showed a higher prevalence of colon cancers versus rectal cancers in the group of patients over 80 years old. Colon cancers represented 87.3% of all colorectal cancers in this age group and 72.4% of all colorectal cancers in patients 80 years old and younger (Fisher’s exact test *p* = 0.02). Presentation as metastatic disease was observed in 11.7% of older and 15.3% of younger patients (Fisher’s exact test *p* = 0.49). Microsatellite instability was more prevalent in older patients in this series (27.3% versus 11.9% in patients younger than 80 years old, Fisher’s exact test *p* = 0.005). A high tumor mutation burden (TMB > 10 mutations/Mb) was also more prevalent in patients over 80 years old (26.9% versus 14% in the younger group, Fisher’s exact test *p* = 0.01). Among the frequently mutated colorectal cancer-associated genes, only *BRAF* mutations showed a higher prevalence in the older patient group (23.9% versus 9.9% in younger patients, Fisher’s exact test *p* = 0.002), while other genes had similar mutation rates in the two groups (Figure 1b). Overall, the most consistent difference observed in colorectal cancers occurring in patients older than 80 years old compared with younger patients is the increased prevalence of right-sided cancers in this age group, and this may at least partially explain the higher prevalence of *BRAF* mutations, which are more frequent in right colon cancers, in older patients.

A genomic colon cancer cohort published by investigators in an international collaboration (the Sidra-LUMC AC-ICAM cohort) included 40 of 348 patients (11.5%) older than 80 years old [18]. In this series that included only colon cancers, patients older than 80 years old had right colon primaries in 60% of cases, while younger patients had right primaries in 51.6% of cases, although this difference did not reach statistical significance (Fisher’s exact test *p* = 0.3). High TMB (>10 mutations/Mb) and high fraction of genome altered (FGA), a marker of chromosome instability, were not different in older and younger patients. Similarly, no significant differences in the prevalence of the consensus molecular subtype (CMS) groups and the prevalence of common colorectal cancer mutations between patients older than 80 and younger patients were observed in the series (Figure 1c) [18].

Overall, from the perspective of molecular alterations, the genomic landscape of colorectal cancer in octogenarians is similar to colorectal cancer in younger patients. Right colon cancers may be more prevalent in octogenarians and older patients. Given the epidemiologic association of right colon cancer with gallstone disease, careful evaluation of older patients with gallbladder symptoms for concomitant colon cancer is warranted [19,20]. The prevalence of metastatic disease at diagnosis is not significantly different in octogenarians compared with younger patients in most series. Molecular alterations influencing treatment considerations should be sought in older patients, as recommended in younger counterparts, as they may pinpoint specific treatment options, such as immunotherapy for microsatellite unstable disease.

## 3. Treatments of Colorectal Cancers in Octogenarians and Older Patients

### 3.1. Surgery

A retrospective series of patients in their 80s and 90s included in an Australian database who underwent surgery for colorectal cancer found that patients with stage III disease and with an ASA (American Society of Anesthesiologists) score of 3 or 4 had higher rates of perioperative complications [21]. Post-operative 90-day and 180-day survival was 98.1% and 93.1% in patients aged 80 to 90 years old and 98.1% and 88.9% in patients over 90 years old. In another series of 237 octogenarians, the post-operative 90-day mortality was 9.3% [22]. Factors associated with increased mortality were increasing age, high ASA score, and performance of the operation on an emergency basis. In a population-based study from four European countries (Belgium, Denmark, the Netherlands, and Sweden), the 90-day mortality of octogenarians undergoing colon cancer resections was between 8% and 15%, and for rectal cancer resections 90-day mortality was between 7% and 11% [23]. The 1-year mortality of octogenarians who underwent surgical resection in an American tertiary center was 37% [24]. Post-operative complication rates including anastomotic leaks and abdominopelvic abscesses were not more frequent in octogenarians but were more often lethal than in younger patients. Age, post-operative complications, higher ASA score, and stage III were associated with higher mortality. In the national database of Germany, surgical resections of colorectal cancers in octogenarians were associated with a post-operative in-hospital mortality of 10.6%, compared with 4.9% in all patients in the database [25]. Although surgical site infection and anastomotic leaks were not more frequent in older patients, associated mortality was increased [25]. Others have also observed that when compared with colorectal cancer patients younger than 50 years old, octogenarians undergoing surgery for colorectal cancer had no increased rates of re-intervention and no differences in the Clavien–Dindo classification of post-operative complications [26]. However, given the increased mortality once complications have occurred, surgical treatment of colorectal cancer in octogenarians and older patients should be discussed with patients and their families in an individualized manner.

Laparoscopic colorectal surgery was associated with oncologic outcomes equivalent to outcomes obtained with open surgery in octogenarians operated on for colorectal cancer [27]. A trend towards decreased post-operative morbidity and a significant decrease in operative blood loss were observed with laparoscopic surgery. In another series evaluating elective laparoscopic surgery for colorectal cancer in octogenarians, overall survival and disease-free survival were improved in patients undergoing laparoscopic resections compared with octogenarians who had open resections [28]. Blood transfusions were fewer and length of hospital stay was shorter with laparoscopic surgeries. Rates of anastomotic leaks and post-operative complications were not statistically different between the two surgical techniques [28]. In a retrospective study from the American College of Surgeons National Surgery Quality Improvement Program database, octogenarians undergoing laparoscopic resections had equivalent post-operative complication rates and survival at 1 month and 1 year compared with patients of the same age undergoing open colectomies, but had significantly reduced length of hospital stay [29]. Laparoscopic procedures also led to lower costs due to lower post-operative pneumonia and urinary tract infection rates [27]. Compared with multiple port surgery, single port laparoscopic surgery further reduced length of hospital stay, with equivalent survival outcomes [30]. Robotic-assisted colectomies were feasible in the over 80 years-old population and were associated with Clavien–Dindo grade III and higher operative complications in 12% of patients [31]. Negative surgical margins were obtained in 91.3% of cases using this technique.

The Japanese Society for Cancer of Colon and Rectum registry data showed that resections of primary cancers with negative margins were performed in similar percentages in patients older and younger than 80 years old [12]. In a study from China, significantly worse morbidity and mortality outcomes were observed in octogenarians who underwent emergency colorectal cancer surgery compared with patients of the same age who had scheduled surgeries [32]. Surgical mortality was ten times higher in emergency operations (30.6% versus 3.1% in scheduled surgeries). The rate of surgical complications was also higher and hospital stays were longer in patients who had emergency operations [30].

Liver metastases in octogenarians were metachronous in 79.4% of cases and synchronous in 20.6% of cases. In matched younger patients, metastases were metachronous in 55.9% of cases [33]. This difference was not statistically significant (*p* = 0.08). Hepatic resection in a series of 71 octogenarians, 35 of whom had mostly metachronous colorectal cancer metastases, was feasible with a mortality rate of 8% and major complications in 24% [34]. Major complications included pulmonary and renal failure, delirium, and ascites and were more frequent in men and patients with prolonged intraoperative hepatic vascular pedicle clamping time. A series of 42 octogenarians from several French centers who underwent major hepatectomies for metastatic colorectal cancer and were matched with 168 patients younger than 80 years old showed that there were no significant differences between older and younger patients in surgical morbidity, reoperation, length of hospital stay, and 90-day mortality [35]. A study from Norway suggested that octogenarians that underwent liver metastasectomies for colorectal cancer metastases had lower survival than age-matched controls without cancer but derived similar survival benefit to colorectal cancer patients in their sixties undergoing liver metastasectomies [36]. In addition, with the adoption of contemporary enhanced recovery after surgery (ERAS) programs, hospital stay and outcomes of hepatectomies for liver metastases in octogenarians were shown to be comparable to those observed in patients aged 70 to 79 years old [33].

In a search for factors predicting post-operative mortality at 30 and 180 days in octogenarians deemed at a clinically acceptable risk to undergo surgical resection, investigators at the Brazilian National Cancer Institute identified high CEA, hypoalbuminemia, and re-operation to be independent predictors of increased mortality [37]. The survival at 30 days of patients with none of these factors was 97.2%, while the survival of those with all three factors was 29.3%. The corresponding survivals at 180 days were 91.8% and 2.5%, respectively [37]. In another study of octogenarians undergoing minimally invasive colorectal surgery, hypo-albuminemia was confirmed as a risk factor for both severe surgical and medical complications [38]. In this study, patients had participated in an ERAS program and their major medical and surgical complications rate was 6.1% [38].

### 3.2. Chemotherapy

Compared with patients aged 65 to 79, older patients in the U.S.A. received chemotherapy less often and had inferior overall and disease specific survival (Table 2) [11]. Among patients in the California Cancer Registry who underwent surgical resection for colon or rectal cancers between 1995 and 2005, 9.4% of octogenarians and 1.6% of nonagenarians with colon cancers received chemotherapy [15]. These percentages were 45.7% in patients younger than 65 years old and 27% in the group of patients 65 to 79 years old. In patients with rectal cancer, 16.3% of octogenarians and 2.4% of nonagenarians received chemotherapy. Rectal cancer patients younger than 65 years old received chemotherapy in 59.1% of cases and patients 65 to 79 years old received chemotherapy in 38.6% of cases [15]. In a more recent series of octogenarians with stage III colorectal cancer registered in the U.S. National Cancer Database from 2006 to 2011, 3483 patients (42.8%) received adjuvant chemotherapy and 57.2% of the patients had surgical resection without adjuvant treatment [39]. Younger octogenarians with fewer comorbidities and N2 disease more frequently received adjuvant chemotherapy.

Adjuvant chemotherapy was given to 47.6% of patients aged 50 to 80 years old and 9.4% of patients older than 80 years old in a series from Boston (Fisher’s exact test *p* < 0.001, Table 2) [13]. In a series from another U.S. academic center, octogenarians, who represented 29.5% of colorectal cancer patients, received chemotherapy much less often for both localized and metastatic disease than younger patients [40].

In other parts of the world, chemotherapy is also utilized less often in patients older than 80 years old than in younger colorectal cancer patients. The Japanese Society for Cancer of Colon and Rectum registry data showed that only 15.9% of patients older than 80 years old with pathologic stage III disease received adjuvant chemotherapy, while 55.5% of younger patients with stage III disease received adjuvant therapy (Table 2) [12]. Although somewhat higher, still only 27.5% of octogenarians with localized disease received adjuvant or neo-adjuvant chemotherapy in a series of colorectal cancer patients treated in Israel [14]. Treatment of localized rectal cancer was adherent to guidelines in only 54% of octogenarians in a retrospective series from the Netherlands [41]. Patients treated according to guidelines had an 18-month survival of 80% versus 56% for octogenarians not treated according to guidelines.

Accurate prediction of geriatric patients at higher risk of developing chemotherapy-associated severe adverse effects could aid in increasing utilization of these therapies in older patients, when indicated, according to their cancer diagnosis and staging. Several tools have been proposed to predict occurrence of adverse effects of chemotherapy in the geriatric population [42,43,44,45,46]. Most of these are not designed specifically for patients above the age of 80 or for colorectal cancer patients, but have been devised by studying populations of geriatric patients above the age of 65 or 70 years old and with all types of primary malignancies. The American Society of Clinical Oncology (ASCO) endorses two tools for the prediction of adverse effects of chemotherapy in geriatric cancer patients [47]. The first tool was proposed and validated by investigators of the Cancer and Aging Research Group (CARG) and stratifies geriatric patients in three risk categories based on a score derived by 11 parameters [42]. The second tool, called the Chemotherapy Risk Assessment Scale for High-Age Patients (CRASH), involves separate calculations for hematologic and non-hematologic toxicities based on several laboratory and clinical parameters, as well as a score for every individual chemotherapy regimen of interest derived from a priori clinical toxicity data with the regimen in question [43]. Both tools are complicated to calculate and rather impractical for the daily oncology clinic. We previously introduced and validated a simpler tool, which performs similarly to the CARG tool but is easier to calculate [45,46]. This predictor, called Index4, is based on only four parameters (ECOG performance status, albumin, creatinine clearance, and stage of the cancer). It should be mentioned, however, that all these tools are not very accurate and further improvements are needed for better prediction in clinical practice of adverse effects and other adverse outcomes of chemotherapy in older patients with cancer.

Similar to chemotherapy, radiation therapy for rectal cancer is under-utilized in older patients. Among patients in the California Cancer Registry who underwent surgical resection for rectal cancers, 15.3% of octogenarians and 5.8% of nonagenarians underwent neo-adjuvant or adjuvant radiation therapy [15]. These percentages were 46.4% in patients younger than 65 years old and 30.9% in the group of patients 65 to 79 years old. In the U.S. National Cancer Data Base (NCDB), a higher percentage of octogenarians had peri-operative radiation [48]. Specifically, 14.9% of the patients older than 80 years old with stage II or III rectal cancer received no treatment and an additional 29.7% had only surgery [48]. The remainder had pre-operative radiation mostly in combination with chemotherapy or less often as a short course without chemotherapy.

## 4. Survival Outcomes and Intensity of Therapy in Patients Older than 80 Years-Old

Advanced age is an adverse prognostic factor for survival in patients with colorectal cancer, observed in multiple studies. In the Japanese Society for Cancer of Colon and Rectum registry, age above 80 was an adverse prognostic factor for overall survival in the multivariate analysis (*p* < 0.001) [12]. In addition, older patients had worse cancer-specific survival rates compared with all groups of younger cancer patients (age 18–49, age 50–64 and age 65–79) across all stages of colorectal cancer. Statistically significant worse overall survival and cancer-specific survival were also observed in patients older than 80 years old compared with younger patients in the series from Israel (log rank *p* < 0.0001 and 0.009, respectively) [14]. Overall survival and disease-free survival were also inferior in older patients with stage II to IV disease compared with younger patients with the same stages. Even for stage I colorectal cancer patients, survival outcomes were numerically inferior in older patients, although not statistically significantly [10].

The benefit for survival of adjuvant chemotherapy in stage III colorectal cancer has been confirmed in randomized trials, which suggest the combination of 5-fluoropyrimidine/leucovorin and oxaliplatin as the recommended regimen based on superior efficacy compared with 5-fluoropyrimidine/leucovorin therapy [49]. However, patients included in this landmark trial were younger than 75 year old, and thus the benefit of combination regimens is not informed by randomized trials in the older population. Patients over the age of 80 years old from the U.S. National Cancer Database receiving adjuvant chemotherapy for stage III colon cancer showed a median overall survival of 61.7 months compared with 35 months for patients who did not receive adjuvant chemotherapy [39]. Among patients who had been offered chemotherapy but refused, overall survival was worse (median 42.7 months) than for patients who accepted and received adjuvant chemotherapy [39]. Similarly, data from SEER covering the period from 2010 to 2015 confirmed the benefit of chemotherapy in stage III and IV colorectal cancer patients over 80 years old compared with patients who did not receive chemotherapy, regarding both overall and cancer-specific survival [50]. For older patients, 5-fluoropyrimidines monotherapy has been proposed as a valid alternative to combination therapy. Specifically, in the population above age 80 with stage III colorectal cancer, single agent fluoropyrimidines may improve relapse-free and overall survival compared with no therapy [51]. In a meta-analysis of adjuvant trials in elderly colorectal cancer patients, defined in the study as older than 65 or 70 years old, no additional benefit for overall survival and disease-free survival was observed with the addition of oxaliplatin to fluoropyrimidine-based chemotherapy [52]. Thus, regimens without oxaliplatin seem to be adequate for patients above the age of 80 and should be the recommended treatment in stage III disease in patients with good performance status.

Regarding patients older than 80 years old with metastatic disease, those who received intensive chemotherapy, defined as at least two cycles of oxaliplation or irinotecan doublets, had a median overall survival of 21 months. This was not different from the overall survival of metastatic patients of younger age who received similar regimens (median overall survival 24.3 months, adjusted hazard ratio 1.29, 85% confidence interval: 0.84–2.00) [53]. Reduced-intensity systemic chemotherapy would increase the percentage of older patients that are candidates for therapy and provide the opportunity for a broader subset of patients above the age of 80 years old to derive benefit with a reasonable adverse-effect profile.

## 5. Prognostic Factors

Given the higher risks of treatments in the group of octogenarians and older patients, prognostic factors for survival outcomes are valuable for better tailoring therapies. In addition to the cancer stage, other clinical, pathologic, and laboratory parameters have been suggested to predict prognostic information in colorectal cancer patients. Metabolic syndrome is a prognostic factor associated with outcomes in colorectal cancer patients across age groups [54,55]. The component of the syndrome associated most consistently with adverse outcomes is glucose intolerance and diabetes [54,56]. Obesity is less consistently associated with prognosis of colorectal cancer patients, with some studies showing an association and others not showing such a predictive value [57,58]. This is related to the fact that obesity is not a linear prognostic factor in colorectal cancer patients. Patients with normal weight have worse prognosis than overweight patients, and obese patients have worse prognosis than their overweight counterparts in several pertinent series. In addition, the implications of central (visceral) obesity are distinct from peripheral obesity, the former being more strongly associated with insulin resistance and metabolic syndrome [59]. In a series of 471 patients aged over 80 years old in Japan, higher Body Mass Index (BMI) above 23 kg/m^2^ was associated with better survival than patients with normal weight and a BMI below 23 kg/m^2^ [60]. In a propensity-score-matched comparison, patients with stage I to III colorectal cancer and a BMI above 23 kg/m^2^ had a 5-year cancer-specific survival of 87.7% and a 5-year overall survival of 78.6% compared with 79.2% and 58.2% in patients with a BMI below 23 kg/m^2^ [60]. Moreover, another Japanese series found that octogenarians with stage 0 to III colorectal cancer and a normal BMI (18.5–24.9 kg/m^2^) had better OS than patients with BMI below 18.5 [61].

Another group of prognostic biomarkers is based on laboratory parameters from the blood, such as hemoglobin, lymphocyte, neutrophil, and platelet counts, and these have been determined to be prognostic factors in colorectal cancer patients of all ages [62,63,64,65]. A composite biomarker consisting of hemoglobin, albumin, lymphocytes, and platelets was studied specifically in the population of patients above 80 years old with localized colorectal cancer and was found to be predictive of survival in a multivariate analysis model [66].

Refinement of molecular prognostic and predictive factors for colorectal cancer has improved outcomes in all age groups, including older patients, by matching therapies with patients most likely to respond. Examples of molecular aberrations which are already used clinically as predictive markers for specific therapies include *KRAS* mutations, which predict lack of benefit from EGFR-targeting therapies, and *BRAF* mutations, for which the benefit of combinations of BRAF inhibitors and EGFR monoclonal antibodies has been shown in second-line metastatic disease treatment [67,68]. Moreover, microsatellite instability-high (MSI-H) cancers benefit from immunotherapy with checkpoint inhibitors [69]. Other molecular aberrations that are less frequent in colorectal cancer but have matched targeted therapies available include HER2 amplifications and NTRK fusions [70,71]. Checking for potentially targetable alterations in patients over the age of 80 years old could offer effective therapies as an alternative to chemotherapy in a sub-set of them. Although targeted treatments are not devoid of adverse effects, they are usually better tolerated than chemotherapy and could be used in some patients with less than optimal performance status.

## 6. Conclusions

Colorectal cancer patients 80 years old and older present management challenges that are unique in this age population [72]. Although disease presentation and the biology of colorectal cancer may differ in some aspects in older patients, most characteristics and the natural history of the disease are not fundamentally different. Perioperative complications and systemic treatment with chemotherapy are more challenging in the older population. In the adjuvant setting, any benefit of chemotherapy is derived from lower-level evidence compared with younger patients. If decided upon, chemotherapeutic monotherapy should be the treatment of choice in octogenarians. A guideline from the French Society for Geriatric Oncology suggests that geriatric metastatic colorectal cancer patients should not be treated with chemotherapy if they have significant comorbidities or functional limitations and should be treated with chemotherapeutic monotherapy of a fluoropyrimidine analogue plus bevacizumab [73]. Chemotherapy combinations are only suggested for patients with symptoms related to the cancer or when the goal is to shrink the tumor for metastatic resection or ablation. Accurate prediction of disease outcomes in the elderly and of the risk of adverse effects of chemotherapy is important for devising effective and tolerable therapeutic plans [44,74]. An improved understanding of the biology and therapy options in daily practice will improve outcomes by better tailoring available options for systemic treatment both in younger patients and the elderly. Moving forward, as new targeted therapies based on the molecular biology of colorectal cancer are introduced, an improved knowledge of the characteristics of the disease in the elderly will provide the impetus for personalized treatment in this age group.

## Figures and Tables

**Table 1:** Presentation characteristics of colorectal cancer in older patients (>80 years old) compared with younger patients. PS: Eastern Cooperative Oncology Group Performance Status; vs: versus; yr: years; yo: years old; *N*: number of patients; NA: not available.

Study	*N* (>80 vs. <80)	Comparator Group	Mean Age (yr)	Comorbidities	Right Location	Grade 3	Stage IV
Lee et al. [10]	133 vs. 596	60–80 yo	83.9 vs. 64.8	83.5% vs. 57% (*p* = 0.01)	32.3% vs. 21.6% (*p* = 0.23)	9.8% vs. 7.4% (*p* = 0.18)	NA
Patel et al. [11]	8643 vs. 24,176	65–80, 50–64, 18–49	85 vs. 42.3, 58 and 72.4	NA	65% vs. 49–58%	23.4% vs. 19.4–25.5%	15% vs. 16–24%
Kotake et al. [12]	3713 vs. 37,138	65–80, 50–64, 18–49	NA	NA	43% vs. 27.8% (*p* < 0.001)	9.1% vs. 6.9% (*p* < 0.001)	14.6% vs. 18% (*p* < 0.001)
Sell et al. [13]	329 vs. 972	50–79	85 vs. 66 (median	63.7% vs. 59.5% (ASA class 3 or 4, *p* < 0.001)	73.3% vs. 55.7% (*p* < 0.001)	NA	6.1% vs. 17.9% (*p* < 0.001)
Goldvaser et al. [14]	175 vs. 175	<80	83 vs. 63	PS > 1: 29% vs. 6.1% (*p* < 0.001)	45.7% vs. 34.3% (*p* = 0.029)	89.3% vs. 80.7% (grade 2 and 3, *p* = 0.025)	20% vs. 22% (*p* = 0.25)

**Table 2:** Treatments and survival outcomes of colorectal cancer in patients older than 80 years old compared with younger patients. vs: versus; yr: years; yo: years old; NA: not available.

Study	Surgery	Chemotherapy	30 Day Mortality	Survival
Lee et al. [10]	No difference in the number of retrieved lymph nodes	Lower rates of chemotherapy administration (adjuvant: 43.3% vs. 92.8%, *p* < 0.001, palliative: 35.8% vs. 89.4%, *p* = 0.01)	NA	OS and DFS decreased overall in the cohort and in stages II to IV
Patel et al. [11]	No difference in the number of retrieved lymph nodes compared with patients 65–79 yo	Lower rates of chemotherapy 8% vs. 24% (65–80 yo), 38% (50–64 yo), and 48% (18–49 yo)	9.3% vs. 4.5%, 3%, and 2.5%	Decreased OS and DSS stage by stage
Kotake et al. [12]	Similar percentages of negative margins obtained	For stage III: 15.9% vs. 55.5%	NA	At 3 and 5 years, OS and CSS worse overall and stage by stage
Sell et al. [13]	No difference in the number of retrieved lymph nodes	Neo-adjuvant: 0.9% vs. 6.8% (*p* < 0.001); adjuvant: 47.6% vs. 9.4% (*p* < 0.001)	3.5% to 4.2% vs. 1.9% to 3.5% stratified according to tumor size (*p* = 0.49)	3-year DFS: 63.8% vs. 59% (*p* = 0.12); Median OS: 4.4 years vs. 6.4 years (*p* < 0.001)
Goldvaser et al. [14]	Surgery or other local interventions less likely (9.7% vs. 65.5%, *p* < 0.0001)	Neo-adjuvant or adjuvant chemotherapy: 27.5% vs. 60.9% (*p* < 0.0001); metastatic chemotherapy: 65.4% vs. 91.2% (*p* < 0.016)	NA	DFS: 68.7% vs. 78.7% (*p* = 0.15); 5-year OS: 38.55 vs. 74.8% (*p* < 0.0001); 5-year CSS: 63.4% vs. 77.6% (DFS: 68.7% vs. 78.7% (*p* = 0.15); 5-year OS: 38.55 vs. 74.8% (*p* < 0.009)

OS: Overall Survival; DFS: Disease Free Survival; CSS: Cancer Specific Survival.

## Data Availability

No additional data beyond data contained in the manuscript are available.
